# Parturition and the Principles and Practice of Obstetrics

**Published:** 1849-09

**Authors:** 


Art. V.—*Parturition and the Principles and Practice of Obstetrics.*—By W. Tyler Smith, M. D., London, Lecturer on Obstetrics in the Hunterian School of Medicine. Lea & Blanchard, 1849. pp. 395. 12 mo.—(From the Publishers. For sale by Jos. Keen, Jr., & Bro.)

This is a singular volume, devoted to the exposition of what the author terms Reflex Obstetrics, which, he informs us, he has been diligently studying for the last seven years. Hitherto, he tells us, Obstetrics has been merely an Art, which his discovery will elevate to the dignity of a Science; to which, at present, we can only answer, "may be yes, and may be no." The Author is particularly savage on Ether and Chloroform; prophesying their speedy expulsion from the lying-in chamber, as, when under their influence, the reflex action of the ovarian nerves induces the ladies to talk bawdy. It is dedicated to Marshal Hall, the Ursa Major of Reflex Action. As it will probably hereafter receive a more extended notice, we forbear any further comments. M.

